# Risk-Based Assessment of 132 kV Electric Distribution Substations and Proximal Residential Areas in the Mangaung Metropolitan Region

**DOI:** 10.3390/ijerph20054365

**Published:** 2023-02-28

**Authors:** Phoka Caiphus Rathebe, Setlamorago Jackson Mbazima

**Affiliations:** 1 Department of Environmental Health, Faculty of Health Sciences, University of Johannesburg, Doornfontein, P.O. Box 524, Johannesburg 2006, South Africa; 2 School of Geography, Archaeology and Environmental Studies, University of the Witwatersrand, Braamfontein, Johannesburg 2050, South Africa; 3 Department of Environmental Sciences, College of Agriculture and Environmental Sciences, University of South Africa, Florida Park, Johannesburg 1709, South Africa; 4 Department of Toxicology and Biochemistry, National Institute of Occupational Health, Division of the National Health Laboratory Service, Braamfontein, Johannesburg 2000, South Africa

**Keywords:** risk compliance, occupational incidents, health and safety, electromagnetic fields, proximity, 132 kV

## Abstract

Annually, an estimate of 2.3 million workers die prematurely due to occupational injuries and illnesses. In this study, a risk assessment was conducted to evaluate the compliance of 132 kV electric distribution substations and proximal residential areas with the South African occupational health and safety Act 85 of 1993. Data were collected from 30 electric distribution substations and 30 proximal residential areas using a checklist. Distribution substations of 132 kV were assigned an overall compliance value of ≥80%, while a composite risk value of < 0.5 was assigned to individual residential areas. The Shapiro–Wilk test was used to check for data normality before multiple comparisons and the Bonferroni adjustment was applied. Non-compliances in electric distribution substations were as a result of poor housekeeping and inappropriate fencing conditions. Ninety-three percent of the electric distribution substations (28/30) scored < 75% compliance on housekeeping and 30% (7/30) were non-compliant (<100%) on fencing. Conversely, there was compliance in the proximal residential areas concerning the substations. Statistically significant differences were found when substation positioning and surrounding infrastructure (*p* < 0.00), electromagnetic field sources (*p* < 0.00) and maintenance/general tidiness (*p* < 0.00) were compared. A peak risk value of 0.6 was observed when comparing the substation positioning with proximal electromagnetic field sources in the residential area. Housekeeping and fencing in the distribution substations must be improved to prevent occupational incidents such as injuries, fire outbreaks, theft and vandalism.

## 1. Introduction

Globally, an estimate of 2.3 million workers die prematurely due to occupational injuries and illnesses on annual basis [1]. Occupational injuries account for 350,000 deaths and occupational illnesses account for two million deaths, while a further estimated five million workers suffer from occupational illnesses due to poor and unsafe working conditions [1,2]. Occupational-related injuries and illnesses are more frequent in developing countries due to poor regulation of occupational health and safety (OHS) and slow advances in technology, monitoring of occupational stressors and disease surveillance [1,3,4]. For example, about 18,000 workers in the Southern African region die from occupational incidents, 13 million more are injured and 67,000 contract occupational illnesses annually [4]. Most occupational injuries, illnesses and premature deaths are preventable; however, control measures are not implemented and maintained [1]. Occupational-related illnesses, injuries and premature deaths have significant negative socioeconomic implications for countries [2]. It is indicated that an average of 4% of the world’s gross domestic product is directly and indirectly lost because of costs associated with compensation, absenteeism, medical expenses, damage to property, restoration and replacement of workers [1,5].

Electric distribution substations are one of the working environments where incidents are likely to occur, particularly if there are no health and safety measures in place. Incidents in electric distribution substations can occur due to design flaws, extreme environmental conditions, oil leaks, lack of maintenance and poor health and safety culture [6]. Common incidents in electric distribution substations include fire outbreaks and explosions, which can affect the surrounding areas [7]. In the Republic of South Africa (RSA), there have been several fire outbreaks and explosions from electric distribution substations resulting in long power outages that have significant negative socioeconomic impacts [8,9,10]. The frequent fires and explosions are a public health concern since most electric distribution substations in RSA are surrounded by residential areas. Moreover, studies have reported that electric distribution substations emit extremely low-frequency electromagnetic fields (ELF-EMFs). Long-term exposure to ELF-EMFs can cause DNA damage, increased oxidative stress, depression, anxiety, stress and poor sleep quality among electric utility workers and people in surrounding residential areas [11,12,13,14]. 

To protect the health and safety of workers, many government departments around the world have developed and promulgated regulations that must be complied with [15]. In RSA, all occupational industries excluding the mining sector are regulated by the occupational health and safety (OHS) Act 85 of 1993. Section 8 of the OHS Act 85 of 1993 stipulates that “every employer shall provide and maintain, as far as reasonably practicable, a working environment that is safe and without risk to the health of his employees”. As such, the employer has a legal obligation to identify hazards and implement interventions and medical surveillance programmes where necessary [16]. Moreover, the OHS Act 85 of 1993 does not only aim to protect the health and safety of workers but also of the surrounding communities. Therefore, the employer also has a legal obligation to protect the health and safety of the surrounding community members. 

Despite the legal requirements of the OHS Act 85 of 1993, many occupational industries in RSA are non-compliant, consequently leading to avoidable occupational incidents. Mrema et al. [4] argued that prevention in occupational environments is not a priority in many African countries. Therefore, there is a need for a preventative approach rather than the previous reactive approach of occupational monitoring and inspection [1]. A proactive approach to protecting the health and safety of employees is conducting a risk assessment to identify risks associated with each stressor in the workplace and have an in-depth understanding of exposure and the consequences [15,17,18]. A risk assessment can be conducted quantitatively or qualitatively depending on the scope, and the results can be used to rate and estimate the probability of harm [19].

In RSA, risk assessment studies have largely focused on petroleum industries [20], healthcare [21], agricultural [22] and mining sectors [23], for the purpose of eliminating both environmental and health effects posed by potential hazards. In the electric utility industry, little is done to quantify the severity of potential risks posed by electrical generation in proximal communities and to electric utility workers. Since electrical substations are located proximal to households, risk quantification is urgently needed, especially in this period of severe electric power cut-offs in South Africa due to load shedding. The majority of substations in the entire RSA have long past their rated operational age and have hardware defects [24]. This presents a significant risk to both electric utility environments and proximal communities. The immediate risks emanating from electrical substations have the potential to affect proximal communities and substation infrastructures [24]. In order to understand the risks of unsafe substations, this study aimed to assess risks in 132 kV substations and the potential impact in proximal residential areas. As observed during the walk-through survey, it is anticipated that the current conditions of the substations present potential risks to the infrastructure and workers. It is also anticipated that such conditions in the substations could pose potential risks to proximal residential areas. In the context of this study, risk is referred to as the probability of an adverse effect or event in a system or population caused under specific circumstances by exposure [25]. The concept of risk definition was applied to conduct a risk assessment in order to evaluate the compliance of 132 kV electricity distribution substations and three surrounding residential areas in the Metropolitan region, Free State Province, RSA. The risk assessment was conducted in line with the requirements of the OHS Act 85 of 1993. To the best of our knowledge, this is the first study to investigate health and safety compliance in 132 kV substations and surrounding residential areas in the RSA. Due to the inherent risk associated with 132 kV substations, it is important to take a proactive approach, thus protecting the health and safety of employees and the nearby community members.

## 2. Materials and Methods

### 2.1. Description of the Study Area

**Figure 1:**
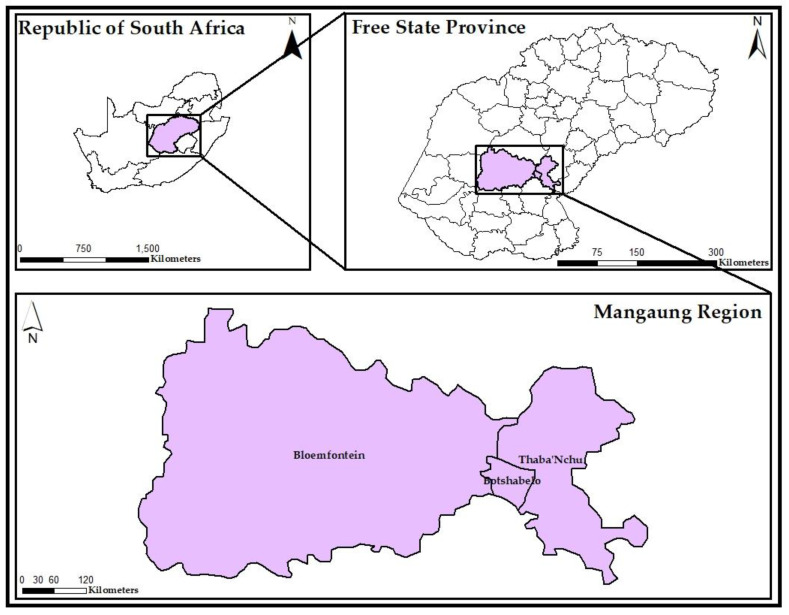
Geographical map of the study area (created using ArcGis 10.9).

This study was conducted in three residential areas in the Mangaung Metropolitan region, Free State Province (Figure 1). The Mangaung Metropolitan region has an area of 628,399 km^2^ and a population size of 747,431. Three residential areas were selected for this study. The three selected residential areas, Bloemfontein (BL), Botshabelo (BO) and Thaba Nchu (TN), have a higher number of electrical substations in the Free State (central) region of RSA. Furthermore, the three residential areas are within 9 km of the evaluated substations. BL has an area of 236.17 km^2^ and a population of 256,185, consisting of equal males and females. BO has an area of 103.98 km^2^ and a population of 181,712, of which 53% are females and the remaining 47% are males. TN is the smallest of the three residential areas, with an area of 36.39 km^2^ and a population size of 70,118, with females and males accounting for 53% and 47% of the population, respectively.

### 2.2. Data Collection

Stratified purposive sampling technique was used and data from the 132 kV electric distribution substations and proximal residential areas were collected from 11 March 2019 to 20 May 2020. The regional electric utility company (CENCLEC) database was used to identify 132 kV electric distribution substations in the study area. A total of 30,132 kV electric distribution substations situated 9 m from the proximal residential areas were evaluated for compliance. Nine of the evaluated electric distribution substations were in BO, six were in TN and fifteen were in BL. Furthermore, 30 residential areas (BN = n15, BO = n9 and TN = n6) were also evaluated for potential risks posed by the proximal electric distribution substations.

Data for this qualitative risk assessment were collected using two observational checklists; one checklist was used for the electric substation and the other for the proximal residential areas. The parameters included in the two used observational tools were adopted from the working conditions regulations in the OHS Act 85 of 1993. The observational checklist used for substations consisted of six different health and safety parameters: (i) housekeeping, (ii) maintenance, (iii) warning signs and access control, (vi) fencing, (v) control room and (vi) registers. The residential area checklists consisted of four parameters: (i) type of residential area, (ii) surrounding infrastructure, (iii) EMF sources and (vi) maintenance. Before conducting the qualitative risk assessment, a walk-through survey was conducted in the electric distribution substations to identify potential electrical hazards and faults. 

#### 2.2.1. Determination of Compliance Values

**Figure 2:**
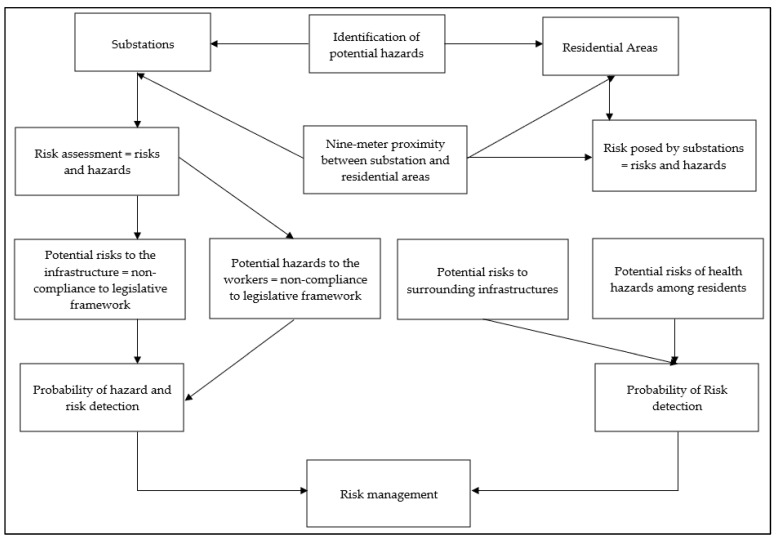
Flow chart for risks identification and management approach.

The electric distribution substation’s health and safety parameters were subjectively assigned compliance percentages and residential areas were assigned risk values based on their health and safety conditions. The allocation of compliance percentage for each health and safety parameter was based on the requirements of OHS Act 85 of 1993. Of the 12 features on housekeeping, nine were stipulated in the OHS Act 85 of 1993 and an overall compliance value of ≥75% was assigned. Warning signs and access control consisted of four features and out of the four, three were required in terms of the act and an overall compliance of ≥75% was also assigned. Control rooms were allocated an overall compliance of ≥67%. This compliance value was based on nine features, of which six are stipulated under the electrical machinery regulations as a requirement. All features on the register, record keeping and fencing are stipulated in the OHS Act 85 of 1993 and a compliance value of 100% was assigned. Any score below the set compliance values was regarded as non-compliance. The overall compliance for individual substations was assigned a total minimum score of ≥80%. Concerning the residential areas, a risk value of <1 indicated compliance, whereas a risk value of ≥1 indicated non-compliance and that the electric distribution substation posed public safety risks for residential areas within a 9 m distance. Furthermore, health and safety compliance for individual residential areas was also evaluated and an overall composite risk mean value of <0.5 was assigned, considering the same distance of 9 m. Figure 2 below demonstrates the process followed to identify potential risks in the substations and proximal residential areas.

#### 2.2.2. Risk Classification and Calculations

##### Substations

Upon assigning the compliance values, risks related to non-compliances were determined using the following formula:

R (risk) = L (likelihood) × S (severity) × D (detection)(1)

where R is considered the risk, L is the likelihood that the risk could occur based on the perceived hazard, S is the severity or impact that could occur as a result of the perceived hazard and D is the probability of detecting the cause of non-compliances. In order to determine the compliance percentages of substations, risk calculation variables were ranked as follow:
L = 1- RareS = 0- noneD = 0- Absolutely uncertain      2- Unlikely      3- Minor      0.9- low      3- Likely      6- Major      1.9- High      4- Very likely      9- Extreme      2.8- Certain      5- Definite      12- Hazardous


##### Residential Areas

To determine the compliance risk values for each proximal residential area, the same formula was used:

R (risk) = L (likelihood) × S (severity) × D (detection)(2)

Since the risk impacts of substations were evaluated in relation to proximal residential areas, the risk calculation variables were assigned new ratings:
L = 0.1- RareS = 0- noneD = 0- Absolutely uncertain      0.2- Unlikely      0.3- Minor      0.9- low      0.3- Likely      0.6- Major      1.9- High      0.4- Very likely      0.9- Extreme      2.8- Certain      0.5- Definite      0.12- Hazardous


### 2.3. Data Analysis

The data were captured on a 2021 version of a Microsoft Excel spreadsheet (Redmond, Washington, DC, USA), where they were cleaned and coded. The IBM statistical package for social sciences (SPSS) version 27 (Chicago, IL, USA) was used to perform all statistical analyses. The normality of the data was checked using the Shapiro–Wilk Test and a Kruskal–Wallis test was used to identify the differences between health and safety parameters on both substations and proximal residential areas. All the tests were assigned a significance level of ≤0.05.

The normality of the data was checked using the Shapiro–Wilk Test, and before performing multiple comparisons (pairwise comparison), the Bonferroni adjustment was applied at ≤0.008 for substation data and ≤0.017 for residential data to avoid the type I statistical error. The mean values for all parameters were used to determine the total compliance values. Derived compliance status was assigned to substations that scored a mean percentage value of ≥80%. A total composite risk mean was also calculated per residential area and a compliance status was allocated per compliance risk value of <0.5 from the checklists.

To provide the risk description and an understanding of the compliance values for each substation, the calculation for housekeeping was carried out as follows:

R = L × S × D(3)

4 × 6 × 2.8 = 67.2

BSL1 compliance value of housekeeping = 67, i.e., it is very likely that the risk of major workplace accidents/incidents would occur, e.g., the risk of fire outbreak due to vegetation or tripping and falling due to loose equipment on the floor. In such events, the probability of detecting the root cause is certain.

To determine the compliance risk values for proximal areas, BLRE1 for EMF sources is used as an example:

R = L × S × D

0.5 × 0.9 × 0.9 = 0.405

BLRE1 compliance risk value for EMF sources = 0.4, i.e., it is definitely likely that the proximity of substations to household EMF sources could have extreme effects either to the households (EMF related health effects) or surroundings (increased EMF exposure levels), and the probability of detecting both the cause and risks is low.

## 3. Results

### 3.1. Compliance of 132 kV Electric Distribution Substations

The results for health and safety parameters of the 132 kV electric distribution substations at the three residential areas are presented in Table 1. From Table 1, it can be noted that on average there was poor housekeeping in all the electric substations. Furthermore, no statistically significant difference was found (*p* > 0.05) when comparing the health and safety parameters for all the substations. However, a statistically significant difference was found when comparing the surrounding infrastructure (*p* < 0.00), EMF sources (*p* < 0.00) and maintenance/general tidiness (*p* < 0.00), which were compared using the Kolmogorov–Smirnov Test. The Shapiro–Wilk test indicated a statistically significant difference when the substations (*p* < 0.0026) and residential areas’ health and safety parameters (*p* < 0.001) were compared.

Table 2 shows the compliance of the electric distribution substations in the three residential areas in the Mangaung region. On housekeeping, 86% (13/15) of the electric distribution substations in BL scored below the compliance value of ≥75%, with only 13% of them (2/15) obtaining 75%. Only 60% (5/15) of the electric distribution substations in BL scored below a significant compliance value of 100% on fencing. However, BL scored a total mean compliance value > 80% in all health and safety parameters (Table 2). Non-compliance was observed for BO and TN substations on housekeeping (<75%). Forty percent (6/15) of substations in BL, 11% (1/9) in BO and 33% (2/6) in TN were not compliant (<100%) with fencing. Only one substation (BOS3) scored below 100% on record keeping. A statistical non-significance was found (*p* = 0.054) when using the Kruskal–Wallis test, indicating no association between the parameters. The Bonferroni test results indicated an association between all substations with regards housekeeping (*p* < 0.00) and record keeping (*p* < 0.00). However, there was no association between substations and (1) warning and access control, (2) control rooms and (4) fencing. With Bonferroni adjustments, the pairwise comparison performed simultaneously between the parameters suggested statistical significances, except the comparison of compliance between control rooms and registers (*p* = 2.29), record keeping and control room (*p* = 5.42), and registers and record keeping (*p* = 9.03).

### 3.2. Compliance of Proximate Residential Environments

Table 3 shows the results for health and safety parameters for the proximal residential areas. The mean risk value was <1 for all the parameters, meaning that the electric substations posed no public health risk to the proximal residential areas. A statistically significant difference was for the position of electric distribution substations (*p* = 0.05); however, no statistically significant difference for EMF sources (*p* = 0.85) and maintenance (*p* = 0.82) was observed.

Compliance results for the three residential areas are presented in Table 4. As shown in Table 4, two residential areas in BL, BLRE1 and BLRE7 scored a significant peak risk value of 0.5 for maintenance and general tidiness. One residential area in the rural area of BL, BLRE3, scored a peak risk value of 0.375, followed by two rural areas in BO, BORE3 (0.375) and BORE6 (0.375), as well as one urban area, BORE5 (0.375), when assessed concerning the risks of surrounding infrastructures. TN had one urban area, TNRE6, with a peak value of 0.6 on EMF sources, followed by BORE1, BORE2 and BORE4 in BO, as well as BRE1 in BL with an equal risk value of 0.4. A total composite risk mean value for all residential areas was <0.5, which indicated compliance in terms of the requirements of OHS Act 85 of 1993. The comparison between all health and safety parameters for residential environments was significantly different (*p* = 0.001) when adjusted for the Bonferroni test. The residential areas were compliant with the proposed risk value scores. The pairwise comparison suggested a statistically significant difference between all composite risk values for residential areas except for compliance between the positioning of substations concerning surrounding infrastructure and EMF sources (*p* = 0.44). The health and safety parameters were compared individually for the three residential areas using the Kruskal–Wallis test and all were statistically non-significant.

## 4. Discussion

The findings of this study suggest a lack of compliance in the 132 kV electric distribution substations and proximal residential areas, which is a health and safety concern for workers and the general public. The main significant non-compliances in the electric distribution substations were poor housekeeping and fencing conditions. Ninety-three percent of the electric distribution substations (28/30) scored <75% compliance on housekeeping and 30% (7/30) were non-compliant (<100%) on fencing. Housekeeping is essential for fire prevention; however, it can easily deteriorate due to a lack of onsite supervision and maintenance [26]. Major factors that contributed to poor housekeeping were the visible weeds, leaking oil from the transformers and loose electrical parts on the ground in the electric distribution substations. It is worth noting that substations are susceptible to weather-related faults [27], and with poor housekeeping, there are potential risks of fire that could completely destroy the infrastructure. In the United Kingdom, McColl et al. [28] found the electrical substations and parts of the electric network to be susceptible to extreme weather conditions. Ideally, electric distribution substations should be free from any weeds or vegetation, including loose objects on the ground, to substantially prevent risks of fire hazards and trip and fall incidents among electric utility workers. Oseni [29] characterized poor maintenance of substations and old electrical infrastructures as factors that lead to electricity outage in Africa. Poor maintenance of substations’ infrastructure and equipment, particularly transformers, can cause fires and explosions, subsequently leading to power outages. Furthermore, oil leaks from transformers can contaminate the soil and nearby water sources, and create fire risks during indirect contact with high-voltage components [30,31]. Similar to the potential risks of poor housekeeping characterized in this study, Minaar et al. [32] suggests the main faults in the electric network of South Africa to be fire outbreaks and pollution.

Explosions and fires in electric distribution substations occur due to numerous reasons; however, the most common cause is a lightning strike that can damage equipment such as transformers, consequently causing excessive damage to the substation components [33]. In cases of fire and explosion, the impact becomes significant if the ground is contaminated with oil, which further makes it difficult to control and extinguish the fire. Therefore, the fencing structures should ideally be made of premoulded concrete slabs covered with anti-thermal material that acts as a barrier to fire and explosion of electrical equipment [34]. In this study, nine substations that scored non-compliance (68%) on fencing had a chain-link fencing structure. Although this type of fencing increases visibility into the substations, it also presents security risks, faulted power lines due to the inexorable movement of humans or animals and attraction of lightning strikes [35,36]. Ward [37] suggested vandalism and theft are the main factors which could lead to hardware failure and various faults in the electric network. In addition to the findings of Ward [37], the current study found non-compliance in the fencing structure. This could possibly lead to theft of hardware and vandalism due to social conflicts [38]. Although the current study represents the compliance conditions of substations in the central region of RSA, Rizzotto [39] confirmed that maintenance of the distribution substations in RSA has been neglected for too long, and this has presented significant challenges in the distribution infrastructures.

Based on the assigned composite risk values, the positioning of electric distribution substations concerning the surrounding infrastructures was compliant with the benchmarked OHS Act 85 of 1993. Mainly, the peak risk value was observed when comparing the substation positioning with proximal sources of (electromagnetic frequency) EMF (overhead power lines, streetlights, electrical junction boxes, etc.). With increased levels of EMFs at the boundaries of substation enclosures [40], the inevitable emission of EMFs from power lines and other electrical sources could significantly increase the exposure levels in proximal residential environments. This could lead to EMF-casual effects in proximal communities [11]. The proximity of substations to other sources of power, such as power lines or electrical junction boxes, intensifies the degree and risks of wildfires during electrical faults. Haces-Fernandez [41] suggests that the proximity of substations or power lines to households creates increased financial risks and potential loss of life. Contrarily, the risk compliance values measured in this study suggest that substations are positioned at a greater distance that would not affect the health and safety of the existing surrounding infrastructures. However, caution should be exercised for new developments not to be positioned closer to substations to avoid potential environmental risks. Moja et al. [42] indicated that positioning of electrical infrastructures proximal to residential areas could have direct impacts on natural resources, fauna, flora, air, water and interrelations with humans. It is therefore essential to implement a risk management strategy. The main objective of this study was to assess risks presented by electric substations found proximal to residential houses. The quantification of risks becomes an incomplete task if a risk reduction strategy is not proposed. Table 5 presents a risk management strategy for the non-compliances observed in this study. Table 6 presents the risk compliance descriptions for risk reduction.

This is the first study in South Africa to assess the compliance of substations using a legislative framework and to determine the risks posed by substations in proximal residential areas. The risk assessment framework used in this study incorporated the risk detection probability to determine whether the assessed potential hazards are likely to be detected. The compliance of electricity infrastructure is best assessed with the requirements of the legislative framework, OHS Act in the case of RSA, since it is the only framework that aims to protect both the occupational setting and its potential risks to the environment. The current study only focused on the 132 kV distribution substation, thus misrepresented potential risks and hazards from other components of the electric distribution network. However, it must be noted that substations are positioned proximal to residential areas, convert a significant voltage of power and could present potential environmental and health risks if not properly maintained.

## 5. Conclusions

Electric distribution substations are hazardous working environments that also pose a risk to proximal residential areas. The findings of this study suggest that poor housekeeping and improper fencing structures could present fire outbreaks, safety accidents, theft and hardware vandalism in the assessed substations. Since Rizzotto [39] suggests that substations in RSA are not properly maintained, the conditions of substations presented by the current study could also be found in other substations across RSA. It is therefore essential to implement occupational health and safety management programmes in all RSA substations in order to eliminate potential occupational risks and environmental hazards. There is an urgent need for quantitative risk assessments in RSA to proactively identify faults that could also lead to economic losses in the electric utility sectors.

In this study, it must be noted that the generalization of the current risk assessment approach to other parts of the world could present a limitation, particularly its application to diverse geographical locations. This approach may be limited by various geographical factors, such as climate change, which often leads to different weather and electricity consumption patterns; age of the substation infrastructure and equipment; local culture and behaviour of the residents; and legislative requirements for a particular area. Therefore, it is imperative for future risk assessment studies to include in their risk quantification approach the various local factors which might influence the assessment of potential risks and hazards presented not only by electrical substations but also power lines and the entire electricity support network.

## Figures and Tables

**Table 1:** Descriptive data for all 123 kV electricity distribution substations.

Parameters	N	Min	Max	Mean	SD	*p*-Value
Housekeeping ≥ 75%	30	33	75	60.67	9.68	0.47
Warning and access control ≥ 75%	30	75	75	75.00	0.00	1.0
Control rooms ≥ 67%	30	89	100	97.07	4.95	0.47
Register (100%)	30	100	100	100.00	0.00	1.0
Fencing (100%)	30	68	100	90.40	14.92	0.47
Record keeping (100%)	30	68	100	98.93	5.84	0.47

N: number of substations, min: minimum, max: maximum, SD: standard deviation.

**Table 2:** Compliance values (%) of distribution substations to health and safety standards.

Substation	Housekeeping	Warning and Access Control	Control rooms	Register	Fencing	Record Keeping	Mean
BLS1	**67**	75	100	100	100	100	88
BLS2	75	75	100	100	**68**	100	86
BLS3	**50**	75	100	100	100	100	88
BLS4	**67**	75	89	100	100	100	89
BLS5	**67**	75	100	100	100	100	90
BLS6	**50**	75	89	100	**68**	100	80
BLS7	**50**	75	89	100	**68**	100	80
BLS8	**58**	75	100	100	**68**	100	84
BLS9	**50**	75	100	100	**68**	100	82
BLS10	75	75	100	100	100	100	92
BLS11	**67**	75	100	100	100	100	90
BLS12	**50**	75	89	100	100	100	86
BLS13	**67**	75	100	100	**68**	100	85
BLS14	**50**	75	100	100	100	100	88
BLS15	**67**	75	100	100	100	100	90
BOS1	**33**	75	100	100	100	100	85
BOS2	**67**	75	89	100	100	100	89
BOS3	**50**	75	89	100	100	**68**	80
BOS4	**67**	75	89	100	**68**	100	83
BOS5	**67**	75	100	100	100	100	90
BOS6	**67**	75	100	100	100	100	90
BOS7	**67**	75	100	100	100	100	90
BOS8	**50**	75	89	100	100	100	86
BOS9	**58**	75	100	100	100	100	89
TNS1	**67**	75	100	100	100	100	90
TNS2	**58**	75	100	100	100	100	89
TNS3	**67**	75	100	100	**68**	100	85
TNS4	**67**	75	100	100	100	100	90
TNS5	**67**	75	100	100	100	100	90
TNS6	**58**	75	100	100	**68**	100	84

Bold values indicate non-compliance.

**Table 3:** Descriptive data for the three residential areas in the Mangaung region.

Parameters	N	Min	Max	Mean	SD	*p*-Value
Position of substations (surrounding infrastructure)	30	0.13	0.38	0.2	0.09	0.05003
EMF sources	30	0.2	0.6	0.24	0.1	0.58
Maintenance/general tidiness	30	0	0.5	0.03	0.13	0. 82

N = number of residential areas, min: minimum, max: maximum, SD: standard deviation.

**Table 4:** Compliance risk values of the three residential areas in the Mangaung region.

Residential Area	Description of Residential Areas	Positioning of Substations: Surrounding Infrastructure (Risk Value < 1)	EMF Sources (Risk Value < 1)	Maintenance/General Tidiness (Risk Value < 1)	Composite Risk Mean Value (Risk Value < 0.5)
BLRE1	Urban	0.125	0.4	0.5	0.34
BLRE2	Urban	0.125	0.2	0	0.11
BLRE3	Rural	0.375	0.2	0	0.19
BLRE4	Rural	0.125	0.2	0	0.11
BLRE5	Urban	0.125	0.2	0	0.11
BLRE6	Rural	0.25	0.2	0	0.15
BLRE7	Rural	0.125	0.2	0.5	0.28
BLRE8	Rural	0.125	0.2	0	0.11
BLRE9	Urban	0.25	0.2	0	0.15
BLRE10	Urban	0.125	0.2	0	0.11
BLRE11	Rural	0.125	0.2	0	0.11
BLRE12	Rural	0.125	0.2	0	0.11
BLRE13	Rural	0.125	0.2	0	0.11
BLRE14	Urban	0.125	0.2	0	0.11
BLRE15	Rural	0.125	0.2	0	0.11
BORE1	Rural	0.125	0.4	0	0.18
BORE2	Rural	0.25	0.4	0	0.22
BORE3	Rural	0.375	0.2	0	0.19
BORE4	Urban	0.25	0.4	0	0.22
BORE5	Urban	0.375	0.2	0	0.19
BORE6	Rural	0.375	0.2	0	0.19
BORE7	Rural	0.125	0.2	0	0.11
BORE8	Rural	0.125	0.2	0	0.11
BORE9	Rural	0.25	0.2	0	0.15
TNRE1	Rural	0.25	0.2	0	0.15
TNRE2	Rural	0.25	0.2	0	0.15
TNRE3	Urban	0.25	0.2	0	0.15
TNRE4	Rural	0.25	0.2	0	0.15
TNRE5	Rural	0.25	0.2	0	0.15
TNRE6	Urban	0.125	0.6	0	0.24

EMF: electromagnetic fields.

**Table 5:** Risk reduction approach for substations and residential areas.

Categories	Compliance Parameters	Required Risk Compliance Levels	Mean Compliance Values	Overall Risk Compliance	Action Required
Substations	Housekeeping	≥75%	60.67	Low	Vegetation/weed and loose equipment on the floor must be removed weekly, and a fire prevention programme must be initiated. Oil leakage checks should be performed monthly as part of the maintenance programme.
	Warning and access control	≥75%	75	High	No action required, unless illuminated warnings can be installed at a very low cost.
	Control rooms	≥67%	97.07	High	No action required, unless a new recording system for all hardware and equipment in the rooms can be implemented at a very low cost.
	Register	100%	100	High	No action required, unless a new recording system can be implemented at a very low cost.
	Fencing	100%	90.40	Medium	Premoulded concrete slabs with anti-thermal material should be installed.
	Record keeping	100%	98.93	High	No action required, unless a new recording system can be implemented at a very low cost.
Residential Areas	Position of substations	<1	0.2	Medium	No developments should be allowed. Sports facilities and preschools with informal structures should be reallocated to new sites.
	EMF sources	<1	0.24	Medium	EMF safety warning sign is urgently required. Monitoring of EMF exposure levels should be undertaken frequently (monthly).
	General tidiness	<1	0.03	High	No action required, unless additional controls can be implemented at a very low cost.

**Table 6:** Risk compliance descriptions.

Compliance Description	Compliance Category
Required compliances for ≥75%	≥75% High
	62–74% Medium
	0–61% Low
Required compliances for 100%	91–100% High
	80–90 Medium
	0–89% Low
Required compliances for ≥67%	67–100% High
	50–66 Medium
	0–49% Low
Required compliances for <1	0–0.1 High
	0.2–0.4 Medium
	0.5–1 Low

## Data Availability

Not applicable.
